# Structural evolution of tunneling oxide passivating contact upon thermal annealing

**DOI:** 10.1038/s41598-017-13180-y

**Published:** 2017-10-16

**Authors:** Sungjin Choi, Kwan Hong Min, Myeong Sang Jeong, Jeong In Lee, Min Gu Kang, Hee-Eun Song, Yoonmook Kang, Hae-Seok Lee, Donghwan Kim, Ka-Hyun Kim

**Affiliations:** 1 Department of Energy and Environment, Graduate School of Energy and Environment, (Green School), Korea University, 02841 Seoul, South Korea; 2 Photovoltaic Laboratory, Korea Institute of Energy Research, 34129 Daejeon, South Korea; 3 Department of Materials Science and Engineering, Korea University, 02841 Seoul, South Korea; 4 KIER-UNIST Advanced Center for Energy, Korea Institute of Energy Research, 44919 Ulsan, South Korea

**Keywords:** Devices for energy harvesting, Surfaces, interfaces and thin films

## Abstract

We report on the structural evolution of tunneling oxide passivating contact (TOPCon) for high efficient solar cells upon thermal annealing. The evolution of doped hydrogenated amorphous silicon (a-Si:H) into polycrystalline-silicon (poly-Si) by thermal annealing was accompanied with significant structural changes. Annealing at 600 °C for one minute introduced an increase in the implied open circuit voltage (V_oc_) due to the hydrogen motion, but the implied V_oc_ decreased again at 600 °C for five minutes. At annealing temperature above 800 °C, a-Si:H crystallized and formed poly-Si and thickness of tunneling oxide slightly decreased. The thickness of the interface tunneling oxide gradually decreased and the pinholes are formed through the tunneling oxide at a higher annealing temperature up to 1000 °C, which introduced the deteriorated carrier selectivity of the TOPCon structure. Our results indicate a correlation between the structural evolution of the TOPCon passivating contact and its passivation property at different stages of structural transition from the a-Si:H to the poly-Si as well as changes in the thickness profile of the tunneling oxide upon thermal annealing. Our result suggests that there is an optimum thickness of the tunneling oxide for passivating electron contact, in a range between 1.2 to 1.5 nm.

## Introduction

Back surface passivation in crystalline silicon solar cells is one of the important key technologies that can achieve high efficiency. A passivated rear contact suppresses back surface recombination, resulting in a high open circuit voltage (V_oc_)^1^. Conventionally, the back surface field (BSF) of the crystalline silicon is formed by aluminum diffusion^2^. The use of conventional BSF has introduced significant recombination at the interface between the metal contact and solar cell^3^. Suggested solutions to the back surface passivation have included the passivated emitter rear locally diffused (PERL)^4^ or the passivated emitter rear contact (PERC)^5^. Both structures have a passivated rear surface and small aperture of local openings at the back contact. However, such local contact concepts not only require additional patterning steps, but also suffer from a fundamental trade-off between passivation (V_oc_) and series resistance (Fill Factor)^6^. In the local contact approach, metallization is typically done by laser ablation on a passivation dielectric layer deposited on the rear surface, and the local contact areas within the passivation layer are opened by laser. The metallization fraction reduces not only the series resistance, but also the passivated surface area. Thus, an excessive metallization fraction also reduces the passivation effect at the rear side.

Another approach to realize passivating contacts is silicon heterojunction by using intrinsic hydrogenated amorphous silicon (a-Si:H), which is also known as heterojunction with an intrinsic thin layer (HIT)^7^. However, the fabrication process of silicon heterojunction solar cells largely relies on a thin film process and is hardly compatible with the conventional crystalline silicon solar cell process.

Tunneling oxide passivating contact (TOPCon) is a structure recently highlighted as it achieved a high conversion efficiency of 25.7%^8^. The TOPCon structure consists of a thin tunnel oxide and a phosphorous (P) doped poly-Si layer contact. The P-doped polycrystalline-silicon (poly-Si) layer can be fabricated by either crystallizing a-Si:H^9^ or by direct deposition of poly-Si using LPCVD^10^. The TOPCon solar cell is a promising candidate for the highly efficient solar cell technology. In particular, the TOPCon structure enables implementation into a conventional solar cell process without cost overruns such as the atomic layer deposition for Al_2_O_3_ passivation and the local contact formation using laser ablation.

A lot of research on the TOPCon passivating contact is ongoing, and there are two different pictures depicting the working principle^11–14^. The direct tunneling of carriers via a very thin oxide thickness is one popular explanation for the carrier selectivity^8,11,12^ while localized carrier transport through pinholes in the interfacial oxide has been proposed based on the practical result of the interfacial oxide breaking up upon thermal annealing^13,14^.

In spite of a lot of research results, a detailed study on the structural evolution of the passivating contact layer stack upon thermal annealing is still missing. In this work, we aimed to study the structural evolution of tunneling oxide electron contact upon thermal annealing at various temperatures. In particular, we examined the evolution of a doped a-Si:H thin film into crystallized poly-Si by thermal annealing with significant phase changes. In addition, the thickness profile of the tunneling oxide was also changed by thermal annealing. Since the thickness of the tunneling oxide plays a crucial role on the carrier selectivity, our results suggest that there is an optimum thickness of the tunneling oxide in passivating tunneling contact structure, which in a range from 1.2 to 1.5 nm for electron contacts. The structural evolution of the TOPCon electron contact upon annealing at different temperatures was analyzed by spectroscopic ellipsometry (SE) and its modeling. The SE measurement and modeling results are further supported by comprehensive analysis using quasi-steady-state photoconductance (QSSPC), secondary ion mass spectrometry (SIMS), and transmission electron microscopy (TEM).

## Results and Discussion

**Figure 1:**
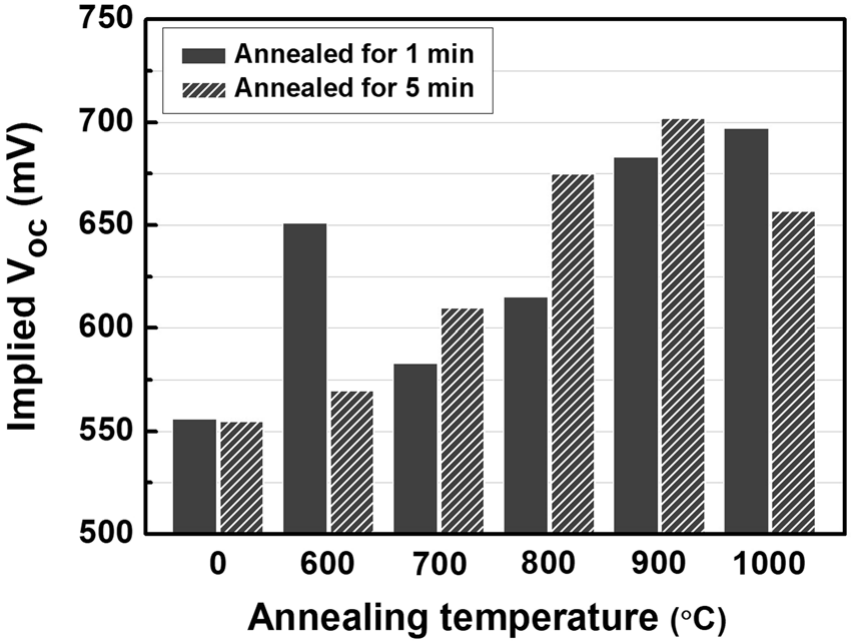
The implied V_oc_ of double side TOPCon electron contacts measured by quasi-stead-state photoconductance annealing at various temperatures and times.

The microstructural and electrical properties of the TOPCon layer stack greatly depends on its annealing condition. The evolution of the microstructure of the TOPCon layer stack plays a crucial role on the solar cell properties such as back surface passivation, carrier selectivity, and carrier transport. Therefore, in this work, the evolution of the microstructure of the TOPCon layer stack upon thermal annealing was studied in detail. Figure 1 shows the evolution of the implied V_oc_ after annealing at various conditions of the TOPCon electron contact fabricated on both sides of a 190 μm thick n-type solar grade wafer substrate. The particular annealing time of one and five minutes was decided by two technological considerations. At first, the annealing time should not be very long in order to prevent the diffusion of phosphorus from the doped a-Si:H into the silicon substrate. Second, the short time of thermal annealing enables the TOPCon contact formation to be implemented into a conventional solar cell manufacture process, such as firing, without additional process steps or extra equipment cost.

The implied V_oc_ of the as-deposited sample was found to be 556 mV, and it showed an increase to 651 mV after annealing at 600 °C for one minute. The implied V_oc_ of the sample decreased to 583 mV after annealing at a higher temperature of 700 °C for one minute, but increased to 615 mV at 800 °C. The implied V_oc_ of the sample continued to increase to 683 mV after annealing at 900 °C and 697 mV after annealing at 1000 °C. It is interesting that for the samples annealed for one minute there was a sudden increase in the implied V_oc_ after annealing at 600 °C, but there was degradation at the higher temperature annealing of 700 and 800 °C for one minute. It is also of note that the implied V_oc_ of the sample annealed at temperatures above 800 °C tended to ramp up until the temperature reached 1000 °C. There was a drastic change in the implied V_oc_ of the samples annealed for longer than five minutes. At 600 °C, the implied V_oc_ of the samples showed a large increase to 651 mV after annealing for one minute. However, further annealing for five minutes led to a drop in the implied V_oc_ of samples to 570 mV. Such drastic changes in the implied V_oc_ of the TOPCon electron contact was tested its reproducibility with more than four types of independent test series using a-Si:H films deposited in different process conditions.

It should also be pointed out that the samples annealed at a higher temperature of 700, 800, and 900 °C showed a continuous increase of their implied V_oc_ upon annealing time. In the case of the sample annealed at 900 °C, the implied V_oc_ of the sample increased to 702 mV. However, the implied V_oc_ of the sample decreased to 657 mV after annealing at a higher temperature of 1000 °C for five minutes. It is necessary to have a more detailed study on the physical origins of the evolution of the implied V_oc_ by the annealing of the TOPCon electron contact.

**Figure 2:**
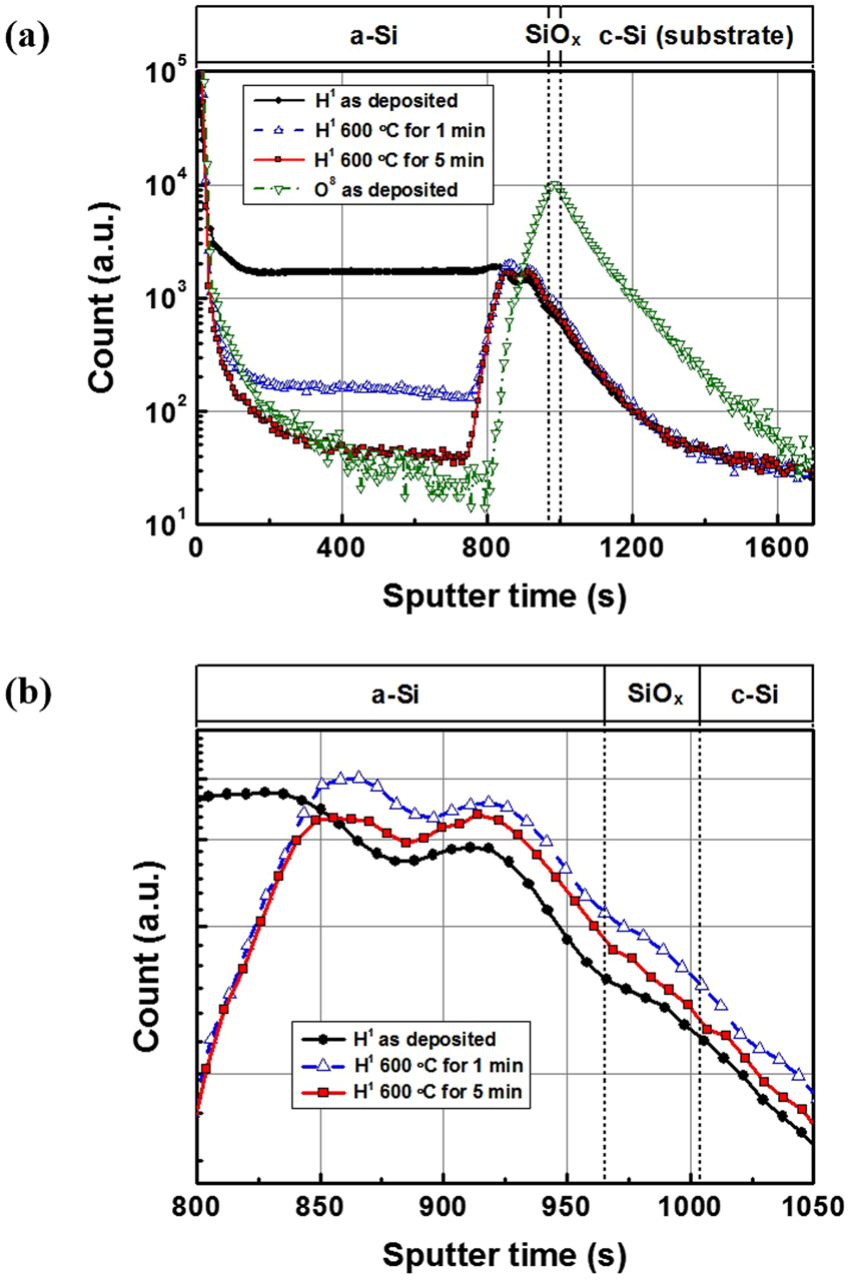
(**a**) The depth profile analysis of the thin film black surface field’s hydrogen concentration measured by secondary ion mass spectrometry at the as-deposited state and after annealing at 600 °C for one and five minutes, respectively, and (**b**) its zoom-in of the interface region between the a-Si:H and SiO_x_ layers.

Figure 2 shows a depth profile analysis of hydrogen concentration of the TOPCon electron contacts at the as-deposited state and annealing at 600 °C for one and five minutes, respectively, by using secondary ion mass spectrometry (SIMS). The SIMS analysis of the elemental composition from the surface of the sample is done by sputtering, and the depth profile analysis can be obtained throughout the sputtering time. The sputtering time in the x axis of Fig. 2 is attributed to the sputter depth (or sample thickness). The measured hydrogen concentration profiles were normalized at a bulk wafer level. At the as-deposited state, the 50 nm thick a-Si:H film shows a hydrogen concentration of 1700 arb. unit. In quantitative translation, this value corresponds to 5.5 × 10^21^ cm^−3^, which indicates the hydrogen content of the a-Si:H film in ordinary cases to be about 10~15 at.%. After annealing at 600 °C for one minute, there is a striking change in that the hydrogen concentration of the a-Si:H film decreased to a level of 160 arb. unit, while the hydrogen concentration at the interface of the a-Si:H/SiO_x_ increased to 950 arb. unit compared to 710 arb. unit in the as-deposited state. After annealing at 600 °C for five minutes, further reduction of the hydrogen concentration in the a-Si:H film, as well as the hydrogen concentration at the interface, was observed to have gone down to the level of 50 arb. unit. As mentioned above, the PECVD deposited a-Si:H usually consists of hydrogen around 10~15 at.%, and the hydrogen effuses out from the film at temperatures above 400 °C^15^. As compared to the hydrogen level detected in the crystalline silicon in Fig. 2, it is believed that all of the hydrogen in the film effused out after annealing at 600 °C for five minutes. In other words, upon annealing at 600 °C, the hydrogen bonded to silicon in thin films became mobile and effused out, and eventually depleted after five minutes of annealing.

There is general consensus that in the case of the PECVD deposited SiN_x_ layer, the firing process that introduces mobile hydrogen gets released, diffuses, and passivates electrically active defects^16^. In spite of the fact that direct observation of hydrogen motions is difficult and the study is limited to indirect analysis through the performance improvement of solar cells, such a beneficial effect of the hydrogen release and interface passivation is generally accepted^17^. One may notice that a large concentration of hydrogen is already detected at the interface, but shows a low implied V_oc_ for the sample of the as-deposited state. There are two reasons why the sample in the as-deposited state shows a low implied V_oc_ despite the high hydrogen concentration at the interface. This is often found in silicon heterojunction technology, where the rearrangement of Si-H bonding changes the surface passivation properties^18^. Even though the changes in the hydrogen content are small, the rearrangement in the Si-H bonding configuration can also result in significant enhancement through thermal annealing.

The second point is the measurement artifact of the SIMS measurement. In spite of a high hydrogen concentration in the interface being detected at the as-deposited state, the hydrogen concentration at the interface could have been hindered by a high hydrogen concentration of the a-Si:H film and the tailing effect. One of the most important types of measurement artifacts during the SIMS measurement is known as cascade mixing. This originates from primary ions striking sample atoms and displacing them from their lattice positions, leading to the homogenization of all atoms within the depth affected by the collision cascade. Lightweight impurity atoms are usually affected and redistributed throughout this “mixing depth” as sputtering proceeds, and the impurity profile will give a deeper distribution, thus introducing the so-called “tailing effect”^19,20^. Therefore, one should be careful in interpreting the hydrogen depth profile of the sample in the as-deposited state. The hydrogen concentration at the interface of the as-deposited state could have been hindered by a high hydrogen concentration in the a-Si:H film and its profile tail.

**Figure 3:**
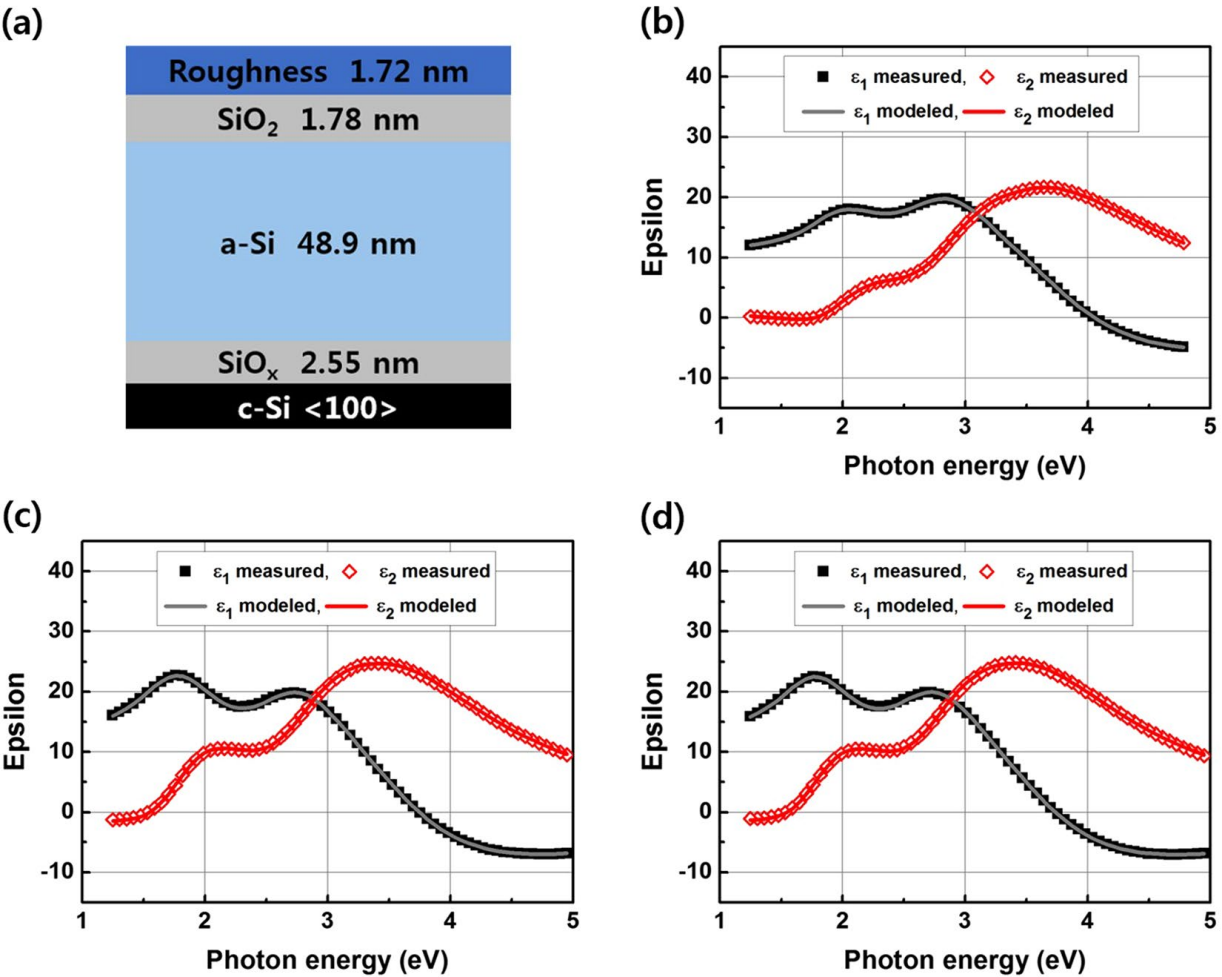
Spectroscopic Ellipsometry (SE) measurement and modeling results of the thin film back surface field before and after annealing at 600 °C. (**a**) Model structure of the as-deposited sample. (**b**) Measured and modeled SE spectra of the as-deposited state. (**c**) Measured and modeled SE spectra of the sample annealed at 600 °C for one minute. (**d**) Measured and modeled SE spectra of the sample annealed at 600 °C for five minutes.

Figure 3(b–d) shows the real (ε_1_) and imaginary (ε_2_) parts of the pseudo-dielectric function of the film measured by SE measurement and the modeling results of the as-deposited sample annealed at 600 °C for one and five minutes, respectively. Table 1 summarizes the Tauc-Lorentz modeling parameters of the results in Fig. 3. Not only the pseudo-dielectric functions of the film measured by SE, but also the material parameters deduced from T-L modeling are presented in this figure. The SE spectra of the samples can be modeled using a layer consisting of T-L dispersion (a-Si:H)^21^ with surface roughness on a Cauchy dielectric (interface oxide). The modeling results reveal significant changes in the material properties upon annealing at 600 °C, while maintaining the absence of crystallization in the doped a-Si:H. One of the most notable changes in the fitting parameters is the optical bandgap (E_opt_), as summarized in Table 1. The E_opt_ of the a-Si:H decreased from 1.68 eV in the as-deposited state to 1.47 eV after annealing for one minute and five minutes, respectively. The E_opt_ of amorphous silicon depends on its hydrogen content, and the E_opt_ of the film decreases as the hydrogen content decreases^22,23^. Therefore, the result suggests that the hydrogen content of the film continued to decrease from the as-deposited to annealed states for one and five minutes, respectively. This is also similar to the SIMS result in Fig. 2, and both results support the idea that annealing at 600 °C can effuse hydrogen. There is another interesting point in the SE modeling that the interface oxide thickness increased after annealing at 600 °C. However, one should be cautious in interpreting the SE modeling that SE measurement is sensitive with refractive index contrast, so the increase in the oxide thickness could be an effect of an additional porous layer formation. In order to cross-verify the SE modeling result, detailed observation was done using TEM cross-section analysis.

**Table 1:** Summary of Tauc-Lorentz modeling parameters of the results in Fig. 3. Interface oxide thickness analyzed by TEM cross-section is also presented.

		As-deposited	600 °C, 1 min	600 °C, 5 min
Tauc-Lorentz modeling parameters	E_opt_ (eV)	1.68	1.47	1.47
	Amp (eV)	190	180	182
	Br (eV)	2.45	2.29	2.27
	E_o_ (eV)	3.69	3.64	3.63
Thickness (nm)	Interface oxide (TEM)	1.5	1.5	1.5
	Interface oxide (SE)	2.55	3.24	3.32
	a-Si:H (SE)	48.9	46.9	47.4
	Surface oxide (SE)	1.78	2.46	2.48
	Roughness (SE)	1.72	6.45	6.94

**Figure 4:**
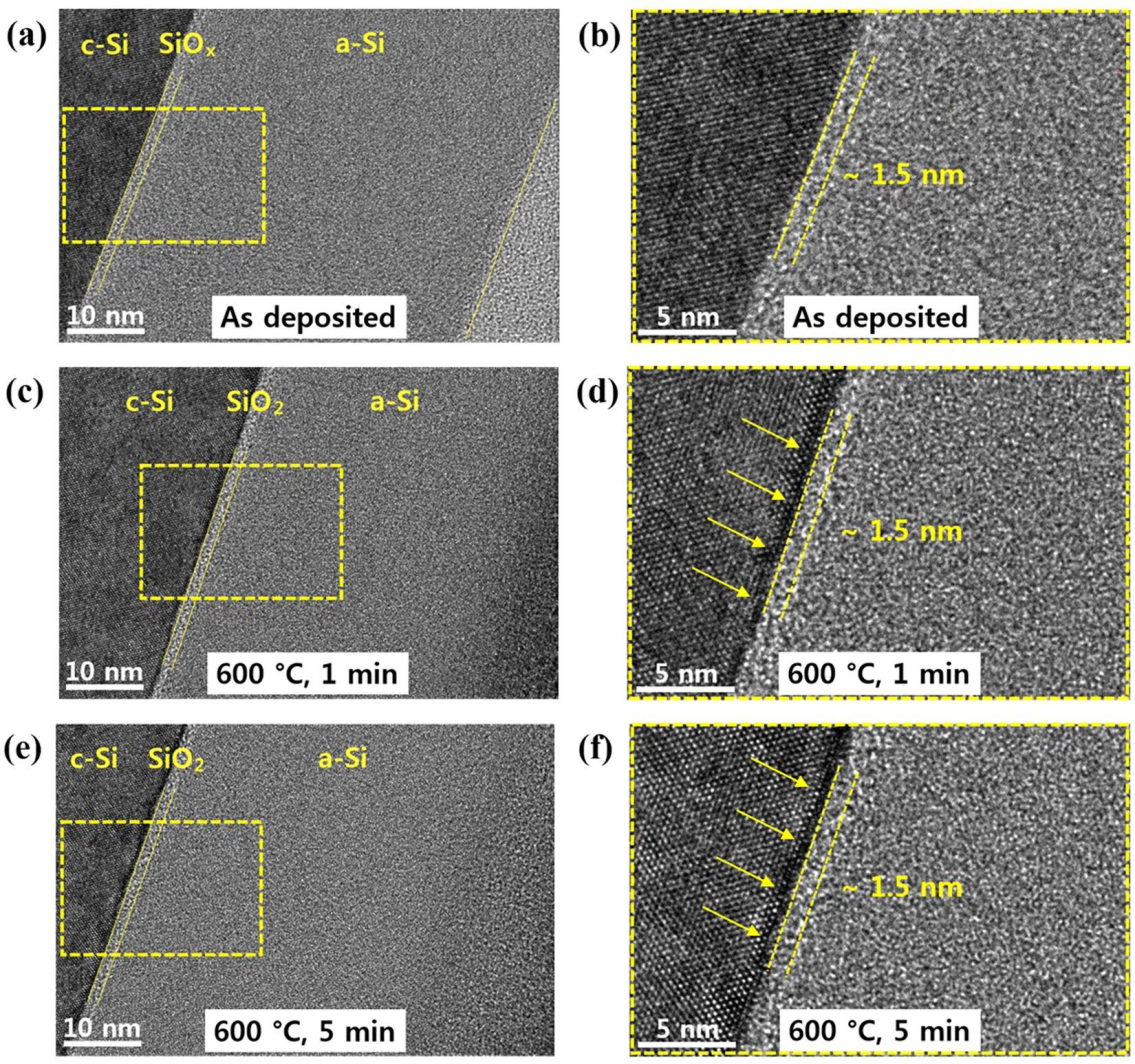
The cross-sectional transmission electron microscopy images of the thin film back surface field in the as-deposited state and annealed at 600 °C for one and five minutes. (**a**) As-deposited. (**b**) Zoom into the interface region (**a**). (**c**) Annealed at 600 °C for one minute. (**d**) Zoom into the interface region (**c**). (**e**) Annealed at 600 °C for five minutes. (**f**) Zoom into the interface region (**e**). Porous interface region (marked by arrows) is observed underneath of c-Si wafer surface, at c-Si/SiO_2_ interface after annealed at 600 °C for both one and five minutes.

Figure 4(a),(c) and (e) shows cross-sectional TEM images of the as-deposited and annealed states at 600 °C for one and five minutes, respectively. Figure 4(b),(d) and (f) shows zoomed images into the interface region of Fig. 4(a),(c) and (e), respectively. The TEM cross-section images verify that all the samples are in an amorphous phase, which is in agreement with the SE modeling result in Fig. 3. However, the interface oxide thickness appears to be unchanged in the TEM cross-section images. Instead, a porous interface region (marked by arrows) is observed underneath the c-Si wafer surface, which is at the c-Si/SiO_2_ interface of the samples after annealing at 600 °C for both one and five minutes. Such a porous region is indeed microstructural damage that is often observed on the c-Si wafer surface after exposure to atomic hydrogen (hydrogen plasma)^24^. The observation of the porous region once again supports the hydrogen effusion during annealing at 600 °C, as discussed above. The TEM cross-section result also suggests that the porous region would have introduced contrast in the refractive index, resulting in changes in the interface oxide thickness in the SE model of Fig. 3. Therefore, it is of note that the increase in the interface oxide thickness after annealing at 600 °C, shown in Fig. 3 and Table 1, should be an artifact that occurred during the optical modeling of the SE result, which originated by the formation of porous microstructural damage after hydrogen effusion. We also made modified optical modeling of the SE results of the samples after annealing at 600 °C, including the porous layer underneath the interface oxide, but the optical model did not show any significant improvement in its reliability because of introduction of additional modeling parameters (void fraction and thickness of the porous layer).

**Figure 5:**
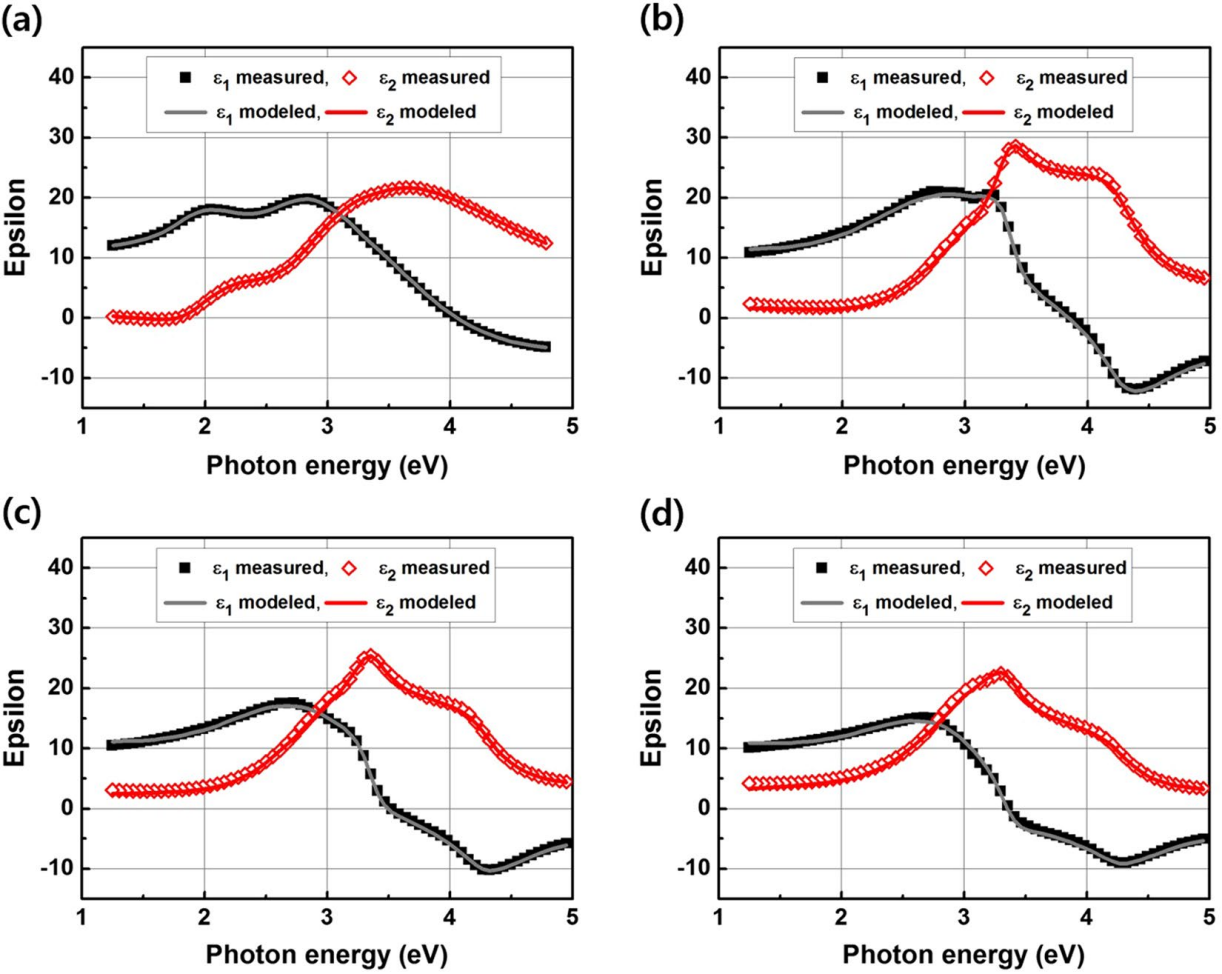
Spectroscopic Ellipsometry (SE) measurement and modeling results of the thin film back surface field before and after annealing at temperatures over 800 °C. The Bruggeman effective medium approximation was used to model the measured spectra. (**a**) Measured and modeled SE spectra of the as-deposited state. (**b**) Measured and modeled SE spectra of the sample annealed at 800 °C for five minutes. (**c**) Measured and modeled SE spectra of the sample annealed at 900 °C for five minutes. (**d**) Measured and modeled SE spectra of the sample annealed at 1000 °C for five minutes.

The material properties of the samples annealed at 800, 900, and 1000 °C, respectively, were also analyzed by SE. Figure 5(b–d) shows the real (ε_1_) and imaginary (ε_2_) parts of the pseudo-dielectric functions of the film measured by spectroscopic ellipsometry measurement and the modeling results of the samples in the as-deposited and annealed states at 800, 900, and 1000 °C, respectively, for five minutes. The measured spectra for the samples annealed at temperatures above 800 °C cannot be modeled with T-L dispersion. The results suggest that the a-Si:H thin films crystallized in a short annealing time at annealing temperatures above 800 °C. The imaginary parts of the pseudo-dielectric function in the SE spectra of the samples annealed at temperatures above 800 °C show two distinct peaks at 3.4 and 4.2 eV, respectively, which represent the first two direct transition energies in the dispersion relation of crystalline silicon^25^. Therefore, the SE spectra of the samples annealed at 800, 900, and 1000 °C were modeled using the Bruggeman effective medium approximation^26,27^. This approach is based on the assumption of the linearity of the mixed phase materials’ optical response, and it enables to define the film structure as expressed in terms of the volume fraction of its constituents. The modeling steps consist of rebuilding the measured pseudo-dielectric function from combining the optical responses of a mixture of known dielectric function materials^26,28^. This approach has been shown to be well adapted to modeling complex multilayer structures consisting of amorphous and nanocrystalline silicon materials. In case of the SE spectrum of material consisting of a small crystallite size, such as nanocrystalline silicon, shows a broadening of these peaks^29^. The measured spectra were modeled by a medium based on the relative fractions of large grain (LG) poly-Si, small grain (SG) poly-Si, and void. Note that a large grain poly-Si refers to an average grain size of about 100 nm while a small grain poly-Si refers to an average grain size of about 10 nm^30^.

**Figure 6:**
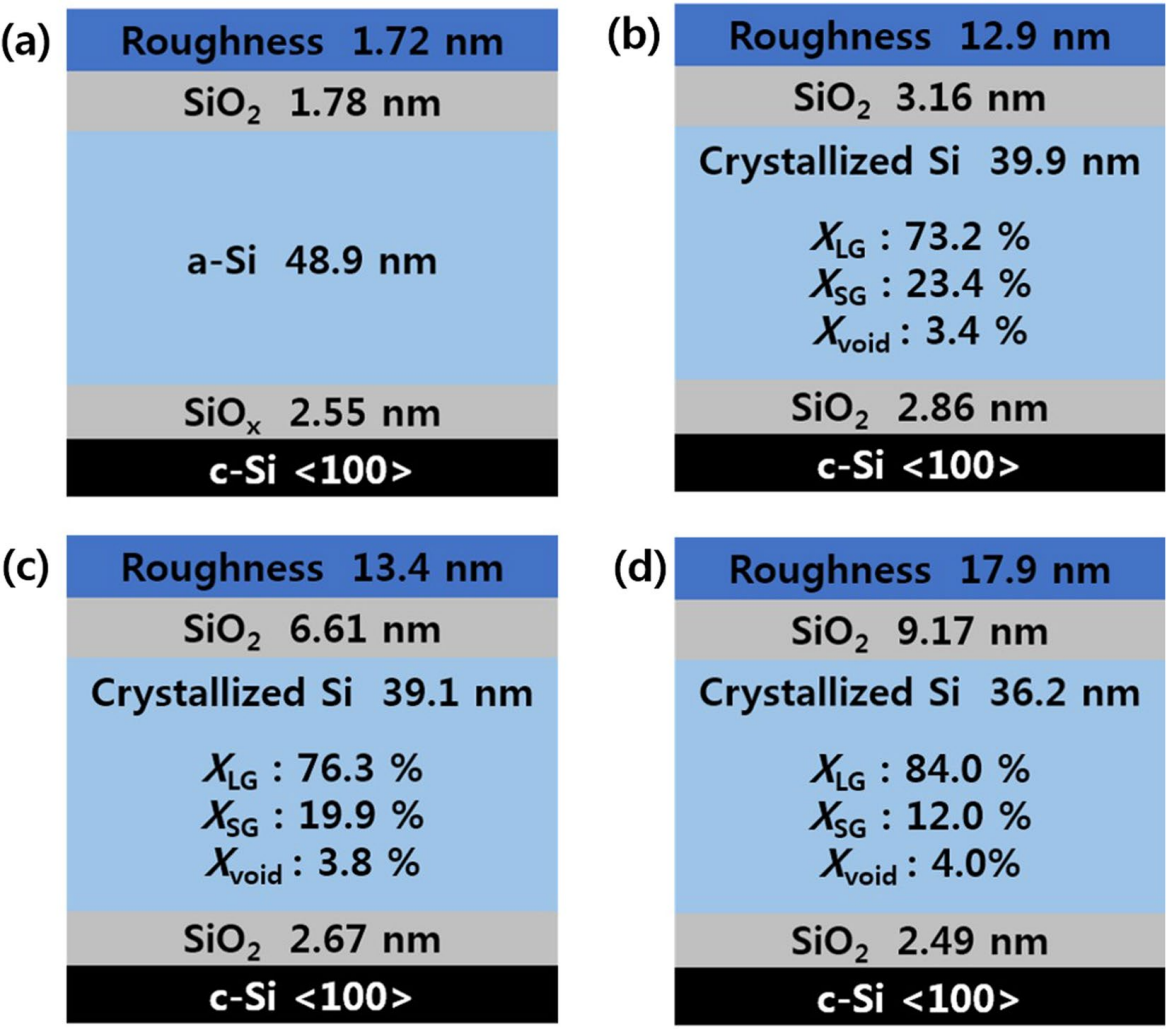
BEMA modeling results of the samples in the (**a**) as-deposited state, annealed at (**b**) 800, (**c**) 900 and (**d**) 1000 °C for five minutes.

Figure 6(a–d) shows the BEMA modeling results of samples in the as-deposited and annealed states at 800, 900, and 1000 °C, respectively, for five minutes. Table 2 summarizes the BEMA modeling parameters of the results in Figs 4 and 5. When comparing the samples annealed at 800 and 900 °C for five minutes, the LG fraction increased from 73.2% to 76.3% and the SG fraction showed a decrease from 23.4% to 19.9%. The LG fraction of the sample showed an increase to 84%, while the SG fraction decreased to 12% after annealing at a higher temperature of 1000 °C for five minutes. In other words, at annealing temperatures of 800, 900, and 1000 °C, both the crystalline volume fraction and crystalline size increased with an increase in the annealing temperature, while the thickness of the surface oxide increased. There is another important point along with the crystallization of the thin film. Furthermore, it is of note that at a higher temperature, the thickness of the tunnel oxide is reduced. In order to observe the thickness of the tunnel oxide in more detail, TEM cross-section measurements on the samples were done.

**Table 2:** Thickness of each layer deduced by the BEMA modeling of spectroscopic ellipsometry spectra and TEM cross section of the results in Figs 5, 6, and 7.

		As-deposited	800 °C, 5 min	900 °C, 5 min	1000 °C, 5 min
Thickness (nm)	Surface silicon oxide	1.78	3.16	6.61	9.17
	a-Si:H/poly-Si	48.9	39.9	39.1	36.2
	Interface silicon oxide (by SE)	2.55	2.86	2.67	2.49
	Interface silicon oxide (by TEM)	1.5	1.4	1.2	1.0

**Figure 7:**
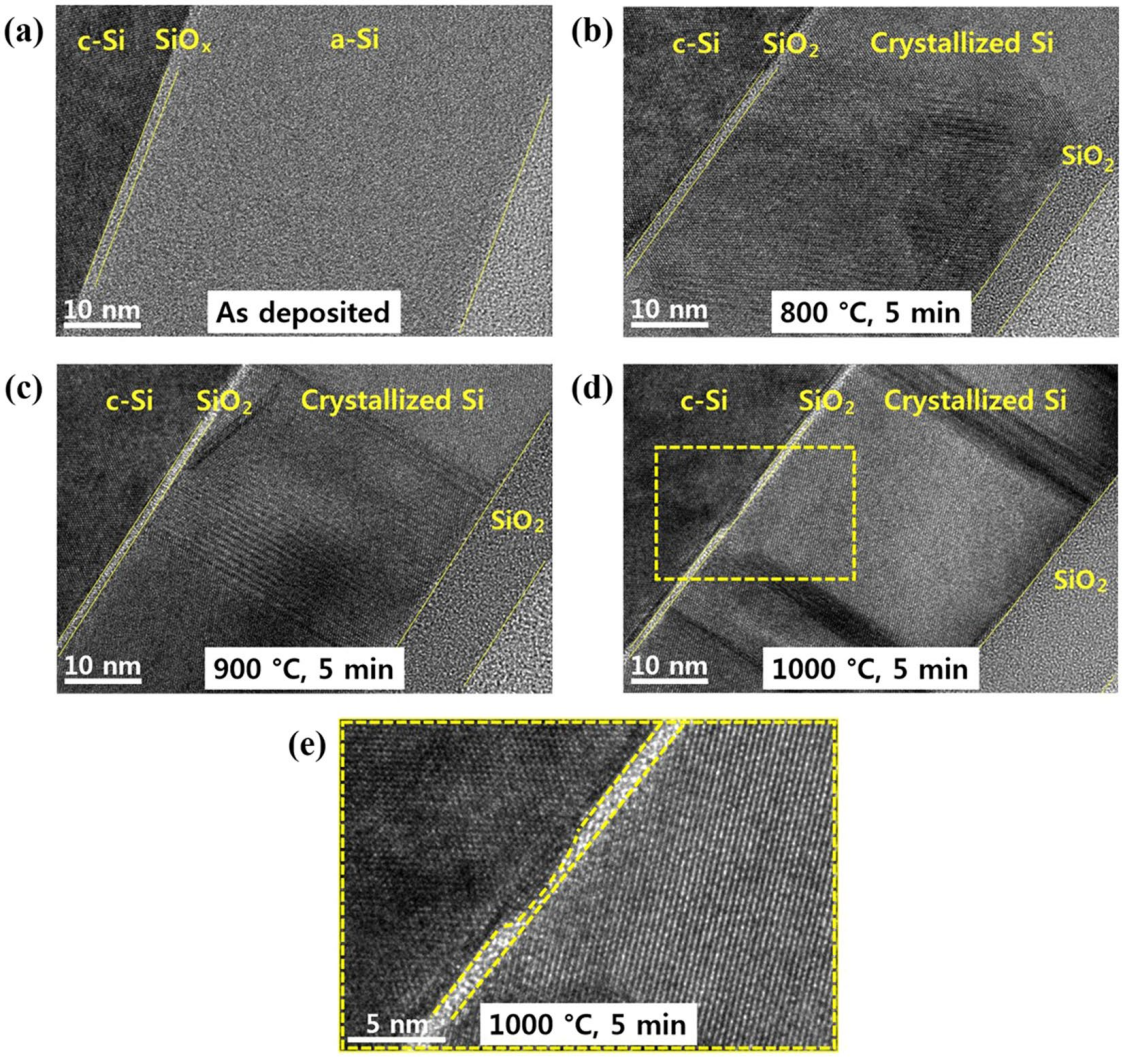
The cross-sectional transmission electron microscopy images of the thin film back surface field in the as-deposited state, annealed at 800, 900, and 1000 °C, respectively, for five minutes. (**a**) As-deposited. (**b**) 800 °C for five minutes. (**c**) 900 °C for five minutes. (**d**) 1000 °C for five minutes, and (**e**) zoom-in of reduced tunnel oxide (**d**).

Figure 7(a) is a cross-sectional TEM image of the as-deposited sample that shows that the structure of the silicon film is clearly amorphous. Figure 7(b)–(d) shows the TEM images of the TOPCon electron contacts annealed at 800, 900, and 1000 °C, respectively, for five minutes. At an annealing temperature above 800 °C, the a-Si:H thin films crystallized. The TEM cross-section images also reveal that the thickness of the tunnel oxide is reduced after the samples are annealed at temperatures above 800 °C. In particular, the tunneling oxide thickness was found to be 1.4, 1.2, and 1.0 nm after annealing at 800, 900, and 1000 °C, respectively. These results are consistent with the SE modeling. Assuming an ideally low interface defect density and good quality oxide, the tunneling oxide thickness and its uniform thickness profile play a crucial role in the carrier selectivity. Tunneling current density through the tunneling oxide depends on the thickness of the tunnel oxide, and can be expressed as below

1 $${I}_{d}=A\,{{\varepsilon }_{ox}}^{2}\cdot \exp (-\frac{B}{{\varepsilon }_{ox}})$$

where *ε*_*ox*_, *A* and *B* are given by

2 $${\varepsilon }_{ox}=\,\frac{{V}_{ox}}{{t}_{ox}}$$

3 $$A=\frac{{q}^{3}}{8{\pi }^{2}h{{\rm{\Phi }}}_{B}}(\frac{m}{{m}_{ox}})=1.54\times {10}^{-6}(\frac{m}{{m}_{ox}})\frac{1}{{{\rm{\Phi }}}_{B}}\,\,\,\,\,\,\,\,\,\,\,[A/{V}^{2}]$$

4 $$B=\frac{8\pi \sqrt{2{m}_{ox}{{\rm{\Phi }}}_{B}^{3}}}{3qh}=6.83\times {10}^{7}\sqrt{\frac{{m}_{ox}{{\rm{\Phi }}}_{B}^{3}}{m}}\,\,\,\,\,\,\,\,\,\,\,\,\,\,\,\,\,\,\,\,[V/cm]$$

with *V*_*ox*_ is the oxide voltage, *t*_*ox*_ is the oxide thickness, q is the electron charge, *h* is Planck’s reduced constant, *Φ*_*B*_ is the effective barrier height, *m*_*ox*_ is the effective electron mass in the oxide, and *m* the free electron mass^31^. Eq. 1 suggests that barrier height (*Φ*_*B*_), effective mass (*m*_*ox*_), and oxide thickness (*t*_*ox*_) are dominant parameters of the tunneling current density.

Since the effective mass of the electrons is lighter than that of the holes in silicon, the tunneling current of electrons would be higher than that of holes^32^. In addition, the tunneling current density decreases as the tunneling length gets longer, which is the thickness of the tunneling oxide in our case. Therefore, the difference in the effective mass of carriers and the thickness of the tunneling oxide layer appear to be major sources of carrier selectivity.

The carrier selectivity of the TOPCon passivating contact can be expressed by the ratio of tunneling probabilities for electrons *T*_*e*_ and for holes *T*_*h*_. Disregarding the barrier shape and effects of image force, the ratio is given by:

5 $$\frac{{T}_{e}}{{T}_{h}}\,\propto \,\frac{\exp ({d}_{ox}\sqrt{\frac{2{m}_{t,e}}{{\hslash }^{2}}{{\rm{\Phi }}}_{e}})}{\exp ({d}_{ox}\sqrt{\frac{2{m}_{t,h}}{{\hslash }^{2}}{{\rm{\Phi }}}_{h}})}$$

Here, *d*_*ox*_ is the oxide thickness, while *m*_*t,e*_(*Φ*_*e*_) and *m*_*t,h*_(*Φ*_*h*_) are the tunneling masses (the barrier height) for the electrons and holes, respectively. *ħ* is the Planck’s constant divided by 2π^11,13^. Eq. 5 suggests that oxide thickness is the main parameter in the ratio of the tunneling probabilities. Considering a 1.5 nm thick oxide, Eq. 5 yields a transmission probability ratio of ratio of electrons/holes about 6.31 while for 2.4 nm thick oxide, a respective value of 19.04 is obtained^13^. The calculation suggests that the carrier selectivity is exponentially proportional to the oxide thickness. There would be an optimum point in the thickness of the tunnel oxide that shows significant carrier selectivity while insuring good carrier transport. Otherwise, the carrier selectivity of the passivated tunnel contact would be insignificant for the tunneling oxide thickness getting too thick or too thin. One can expect that the carrier selectivity in the TOPCon passivating contact would disappear if the oxide thickness is too thin, as predicted in the numerical simulation by Steinkemper *et al*.^11^. In the case of a too thick tunneling oxide, nevertheless the carrier selectivity increases, the tunneling current of both the electrons and holes would be simultaneously suppressed. Thus, the carrier transport of both electrons and holes would be reduced in a thicker oxide^11^. Regarding that the thickness of the tunneling oxide gradually decreases upon a higher annealing temperature, e.g., 800, 900 and 1000 °C, the tunneling current density through the tunneling oxide would vary after thermal annealing at various temperatures.

In our work, the optimum thickness of the tunneling oxide was also found to be in between 1.2 to 1.5 nm observed by TEM, which is also consistent with the results found in the literature elsewhere. Both the numerical simulation and experimental results suggest that the ideal oxide thickness for the TOPCon electron contact should be around 1.5 nm^8,11–13,33^. Last, but not least, in the case of high temperature annealing at 1000 °C, the thickness profile of the tunnel oxide became rough. The rough profile of the tunnel oxide shown in Fig. 7(e) suggests that at a high annealing temperature annealing of 1000 °C, the stoichiometry of tunnel oxide is broken and forms silicon suboxide^12^. The locally reduced tunnel oxide introduces direct contact between the c-Si wafer substrate and n+ poly-Si, transporting both electrons and holes that deteriorates the carrier selectivity of the TOPCon passivating contact.

## Conclusion

In this work, we studied the structural evolution of TOPCon passivating contact upon thermal annealing. We demonstrated that the implied V_oc_ of the TOPCon electron contact depends on the hydrogen motion and the crystallization of the doped a-Si:H. After annealing at 600 °C for one minute, the implied V_oc_ showed a significant increase due to the hydrogen motion, thereby resulting in the chemical passivation of electrically active defects that originated in the effusion of hydrogen from doped a-Si:H. However, at further annealing at 600 °C for five minutes, the implied V_oc_ dropped again because all of the hydrogen was effused out and depleted. Therefore, the changes in the implied V_oc_ after annealing at 600 °C for one minute may be difficult to implement into real devices.

At an annealing temperature above 800 °C, the crystallization of the a-Si:H formed P doped poly-Si. Spectroscopic ellipsometry analysis revealed that the grain size of the crystallized poly-Si increased by annealing at a higher temperature, which further resulted in an increase in the implied V_oc_. After annealing at 1000 °C for five minutes, the implied V_oc_ of the sample decreased. TEM analysis revealed that the decrease is because of a reduced thickness of the tunnel oxide, which introduces the deteriorated carrier selectivity of the TOPCon structure.

## Methods

TOPCon electron contact was fabricated on n-type, 200 μm thick ohm∙cm solar grade wafers with a resistivity of 0.6 ohm∙cm for measuring the implied open circuit voltage (implied V_oc_) upon thermal annealing. 650 μm thick semiconductor grade wafers with a resistivity of 3 ohm∙cm were also used for measurements by spectroscopic ellipsometry (SE), secondary ion mass spectrometry (SIMS), and transmission electron microscopy (TEM) after thermal annealing. The saw damage removal (SDR) of the solar grade wafers was done using KOH solution and deionized water at 80 °C. About 10 μm of SDR was etched on both sides of the damaged wafer surfaces. All wafers were cleaned using the following sequence: 10% HF dip, deionized water + H_2_O_2_ + NH_4_OH (RCA1) at 80 °C for 10 min, deionized water + H_2_O_2_ + HCl (RCA2) at 80 °C for 10 min, and 10% HF dip. Then, a thin silicon oxide was grown using the nitric acid (HNO_3_) solution. The temperature and time for the wet chemical oxidation process were set to be 120 °C and 15 minutes, respectively. The thickness of the silicon oxide layer was found to be about 2.5 and 1.4 nm, deduced by the SE optical model and TEM, respectively.

On the thin oxide layer, hydrogenated amorphous silicon (a-Si:H) thin films were deposited by the capacitively-coupled-plasma (CCP) radio-frequency (RF, 13.56 MHz) glow discharge PECVD method at substrate temperatures ranging from 175 to 200 °C. P doped a-Si:H films were deposited under carefully controlled plasma conditions using hydrogen-diluted silane gas mixtures. The silicon films deposited under such conditions show various controllable material properties. For example, the silicon films can consist of a small fraction of silicon nanocrystals, which work as seeds for the crystalline growth^34–38^. In this work, our a-Si:H layers were deposited at a p∙d product (pressure × inter-electrode distance) ranging from 1 to 3 Torr∙cm and a RF power density in a range of 30 to 100 mW/cm^2^.

After the a-Si:H deposition process, the thermal annealing of the wafers was done at temperatures ranging from 600 to 1000 °C for one and five minutes, respectively, in a quartz tube furnace under a nitrogen atmosphere. At an elevated temperature above 600 °C, a-Si:H was crystallized in a solid phase^39^.

The implied V_oc_ represents the potential V_oc_ of the solar cell, and it can be deduced by using the carrier concentration under illumination^40^:

6 $$Implied\,{V}_{oc}\,=\,\frac{kT}{q}ln(\frac{np}{{{n}_{i}}^{2}})$$

where q is the unit charge, k is the Boltzmann constant, T is the temperature, n_i_ is the intrinsic carrier concentration, n is the electron concentration, and p is the hole concentration. After annealing, the implied V_oc_ of the solar grade wafer was determined by the quasi-steady-state photoconductance (QSSPC) method using WCT-120 made by Sinton instruments.

Semiconductor grade wafers were used for secondary ion mass spectrometry (SIMS) measurement and SE analysis. SIMS is a technique used to analyze the composition of solid surfaces and thin films by sputtering the surface of the specimen with a focused primary ion beam and collecting and analyzing the ejected secondary ions. The hydrogen profiles of the TOPCon electron contact upon different thermal annealing conditions were analyzed by an IMS-6f magnetic sector SIMS from the CAMECA instrument. All wafers were measured with the following analysis conditions: primary ion = Cs+; impact energy = 15 keV; current = 10 nA; analysis area = 30 μm(Φ).

Spectroscopic ellipsometry (SE) is a non-destructive test method used to analyze the optical properties of the sample. It can also deduce various information like the film thickness, crystallinity, and bandgap, depending on the modeling method^41^. The measured SE spectra of the TOPCon electron contact annealed at various temperatures were analyzed by the Tauc-Lorentz (T-L) dispersion model. The ε_2_ of T-L dispersion can be expressed as below^42^:

7 $${\varepsilon }_{2}(E)=0,E\le {E}_{g}$$

8 $${\varepsilon }_{2}(E)=\frac{A{E}_{0}C{(E-{E}_{g})}^{2}}{{({E}^{2}-{E}_{0}^{2})}^{2}+{C}^{2}{E}^{2}}\cdot \frac{1}{E},\,E > {E}_{g}$$

where E_g_ is the optical band gap, A is the amplitude, E_0_ is the peak-to-peak transition energy, and C is the broadening term. Since T-L dispersion considers a single transition in the direct bandgap material, the crystallized poly-Si after thermal annealing cannot be analyzed by T-L dispersion. The poly-Si samples were analyzed using Bruggeman effective medium approximation (BEMA). Analysis using BEMA is widely used to determine the volume fraction of mixed phase materials such as nano- and micro- crystalline materials^30^.

The detailed microscopic structure of the samples was also analyzed using the cross-section measurements from the transmission electron microscopy (TEM). The sample for the cross-section was prepared using a focused ion beam.
