# Author Correction: Structural evolution of tunneling oxide passivating contact upon thermal annealing

**DOI:** 10.1038/s41598-022-05626-9

**Published:** 2022-01-21

**Authors:** Sungjin Choi, Kwan Hong Min, Myeong Sang Jeong, Jeong In Lee, Min Gu Kang, Hee-Eun Song, Yoonmook Kang, Hae-Seok Lee, Donghwan Kim, Ka-Hyun Kim

**Affiliations:** 1 Department of Energy and Environment, Graduate School of Energy and Environment, (Green School), Korea University, 02841 Seoul, South Korea; 2 Photovoltaic Laboratory, Korea Institute of Energy Research, 34129 Daejeon, South Korea; 3 Department of Materials Science and Engineering, Korea University, 02841 Seoul, South Korea; 4 KIER-UNIST Advanced Center for Energy, Korea Institute of Energy Research, 44919 Ulsan, South Korea

Correction to: *Scientific Reports* 10.1038/s41598-017-13180-y, published online 16 October 2017

The original version of this Article contains errors in Equation 5 where a negative sign is omitted.

Equation 5:

$$ \frac{{T_{e} }}{{T_{h} }} \propto \frac{{\exp \left( {d_{ox} \sqrt {\frac{{2m_{t,e} }}{{{\hslash }^{2} }} \Phi_{e} } } \right)}}{{\exp \left( {d_{ox} \sqrt {\frac{{2m_{t,h} }}{{{\hslash }^{2} }} \Phi_{h} } } \right)}} $$

should read:

$$ \frac{{T_{e} }}{{T_{h} }} \propto \frac{{\exp \left( { - d_{ox} \sqrt {\frac{{2m_{t,e} }}{{{\hslash }^{2} }} \Phi_{e} } } \right)}}{{\exp \left( { - d_{ox} \sqrt {\frac{{2m_{t,h} }}{{{\hslash }^{2} }} \Phi_{h} } } \right)}} $$

